# Welding Fumes, a Risk Factor for Lung Diseases

**DOI:** 10.3390/ijerph17072552

**Published:** 2020-04-08

**Authors:** Maria Grazia Riccelli, Matteo Goldoni, Diana Poli, Paola Mozzoni, Delia Cavallo, Massimo Corradi

**Affiliations:** 1 Department of Medicine and Surgery, University of Parma, via A. Gramsci 14, 43126 Parma, Italypaola.mozzoni@unipr.it (P.M.);; 2 Centre for Research in Toxicology (CERT), University of Parma, via A. Gramsci 14, 43126 Parma, Italy; 3 INAIL Research, Department of Occupational and Environmental Medicine, Epidemiology and Hygiene, via Fontana Candida 1, 00078 MontePorzio Catone (Rome), Italy; d.cavallo@inail.it; 4 University Hospital of Parma, via A. Gramsci 14, 43126 Parma, Italy

**Keywords:** welding fumes, particles, oxidative stress, inflammation, lung diseases

## Abstract

(1) Background: Welding fumes (WFs) are composed of fine and ultrafine particles, which may reach the distal airways and represent a risk factor for respiratory diseases. (2) Methods: In vitro and in vivo studies to understand WFs pathogenesis were selected. Epidemiological studies, original articles, review, and meta-analysis to examine solely respiratory disease in welders were included. A systematic literature search, using PubMed, National Institute for Occupational Safety and Health Technical Information Center (NIOSHTIC), and Web of Science databases, was performed. (3) Results: Dose, time of exposure, and composition of WFs affect lung injury. Inflammation, lung defense suppression, oxidative stress, DNA damage, and genotoxic effects were observed after exposure both to mild and stainless steel WFs. (4) Conclusions: The detection of lung diseases associated with specific occupational exposure is crucial as complete avoidance or reduction of the exposure is difficult to achieve. Further studies in the area of particle research may aid the understanding of mechanisms involved in welding-related lung disease and to expand knowledge in welding-related cardiovascular diseases.

## 1. Introduction

The American Welding Society defines welding as “a metal joining process wherein coalescence is produced by heating to suitable temperature or by the application of pressure with or without the use of filler metal” [1]. Different types of welding have been identified in commercial use. Among these, electric arc welding is the most common; in electric arc welding, heat fusion (temperature above 4000 °C) is produced when electricity passes through a gas between two electrical conductors [2]. Table 1 shows different types of electric arc welding. When the electric welding arc is struck between electrode and base metal in air or inert gas, the metal vapor, having evolved at a very high temperature, is then cooled down in the gas stream and condenses to form fumes [3]. Welding fumes’ (WFs’) composition is variable, since the main fume components derive from electrode and filler wire, and from fluxes wherever used. Moreover, base metal, shielding gases, and paint or surface coating may all further contribute to fume formation. Fumes are formed when vaporized metal comes into contact with oxygen, producing metal oxides which then condense and form fumes. Shielding gases may also be added to minimize oxidation. Aluminum (Al), cadmium, cobalt, chromium (Cr), copper (Cu), fluorides, iron (Fe), lead, manganese (Mn), magnesium, molybdenum, nickel (Ni), silica, titanium, and zinc (Zn) are all metal oxide particles that can be present in metal fumes. Toxic gases are also generated during common arc welding processes, such as ozone (O_3_), nitrogen oxides (NO_x_), carbon dioxide, and carbon monoxide. Fumes which are generated from stainless steel (SS) electrodes usually contain Fe, Cr, Mn, and Ni, whereas mild steel (MS) WFs contain 80% Fe, with small amounts of Mn. SS welding presents added occupational hazards compared to MS due to exposure to hexavalent chromium (Cr VI) and Ni, which are two known carcinogens. Welding fume generation rate depends on welding processes. In submerged arc welding (SAW), gas metal arc welding (GMAW), and gas tungsten arc welding (GTAW or TIG) shielding gases reduce oxidation that may occur during the welding process with lower fume emission compared to manual metal arc welding (MMAW) and to flux-cored arc welding (FCAW). However, in GMAW and GTAW shielding gas can intensify ultraviolet radiation leading to photochemical formation of gases such as O_3_ and NO_x_ [4]. 

International Agency for Research on Cancer (IARC) has recently classified both MS and SS WFs as group I carcinogens [5]. It is known that WFs can cause also nonmalignant lung diseases. Additionally, occupational exposure to particle and WFs has been associated with an increased risk of cardiovascular disease, and fine particles (aerodynamic diameter < 2.5 micrometer μm) seem to play a key role [6]. 

The aim of this systematic review of the literature was to understand the toxicological mechanisms responsible for malignant and nonmalignant lung diseases. Moreover, it would be important to know WFs components that are responsible for lung damage and the exposure doses to which the different respiratory diseases occur. 

This review focused on welding related lung diseases, although we recognize the importance of welding as a risk for cardiovascular diseases, too [7,8].

The review is structured into the following parts:

- Welding particle characteristics and the respiratory tract.
- Description of in vitro, in vivo, and in human studies, which addressed pathological mechanisms of WFs.
- Welding-related lung disease.
- Biological monitoring program.

## 2. Materials and Methods

A systematic literature search, using PubMed, National Institute for Occupational Safety and Health Technical Information Center (NIOSHTIC), and Web of Science databases, was performed by two authors with the terms and phrases: “Welding fumes” or “arc welding” and “inflammation” or “oxidative stress” or “DNA damage” or “occupational disease” or “pneumonia” or “occupational asthma” or “lung function” or “Chronic Obstructive Pulmonary Disease (COPD)” or “chronic bronchitis” or “cardiovascular risk” or “lung cancer” or “exhaled breath”. We included original articles, case series, review, and meta-analysis published in the English language until 2019. Abstracts were scanned for potential relevance by the same two authors. The literature was also searched for regulations related to WFs, Cr VI, and Ni in the occupational environment. This search included the National Institute for Occupational Safety and Health (NIOSH), the American Conference of Governmental Industrial Hygienists (ACGIH), the Occupational Safety and Health Administration (OSHA), and the Scientific Committee on Occupational Exposure Limits (SCOEL). Particular importance was given to the articles that explained the generation and deposition in the respiratory tract of WFs particles. Moreover, a critical review of the literature on respiratory diseases caused by WFs was carried out. In vitro, in vivo, and human studies was also included. Overall, we selected 195 citations. In this review, we tried to synthesize a large and complex topic, finally providing indications on old and new prospects for primary and secondary prevention in welders.

## 3. Results

### 3.1. Particle Characteristics and Distribution in the Respiratory Tract

WFs are composed of fine and ultrafine particles (aerodynamic diameter: 0.01–1 µm), which are highly likely to be deposited in terminal bronchioles or alveoli, since most of the primary particles in different aerosols have diameters between 0.005 and 0.04 µm. In many cases, particles tend to aggregate to form primary particle chains, thus changing their size and weight. The number concentration of small-diameter particles appears to markedly decrease as residence time increases, since smaller particles are scavenged by larger particles through coagulation [9]. Dasch et al. showed a bimodal size particle distribution in six welding processes [10], whereas Zimmer et al. found a multimodal distribution of welding aerosol [9]. In GMAW, 15, 120, and 480 seconds after the initial welding burst, particles of 90–246, 104–264, and 200–352 nm diameter were found, respectively [9].

**Figure 1:**
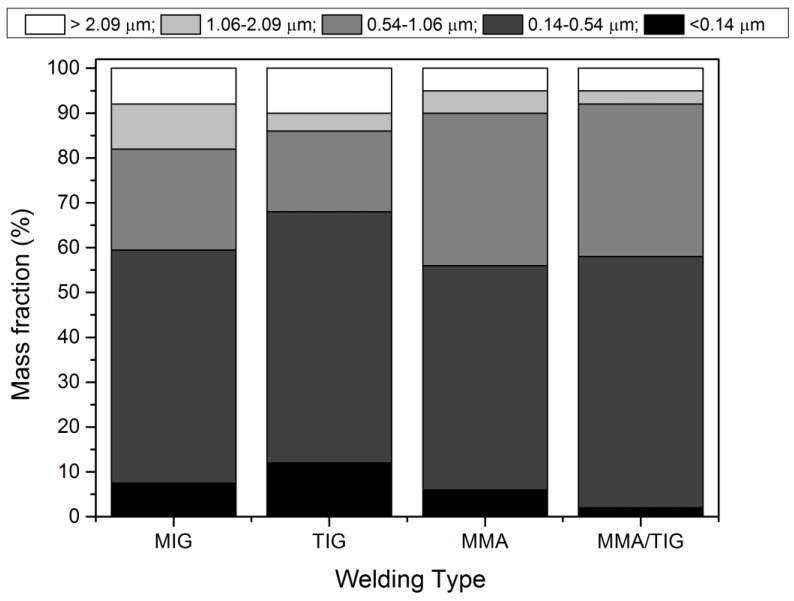
A representation of the possible particle size distributions of welding processes (Berlinger et al. [3]). MIG: metal arc inert gas; TIG: tungsten inert gas; MMA: manual metal arc.

Particle sizes in WFs depend on the welding process utilized. Brand et al. observed count median diameters of about 150 nm for MMAW, 100 nm for metal arc active gas (MAG), 100–150 nm for different metal arc inert gas (MIG), and <50 nm for GTAW and resistant spot welding [11], in agreement with previous observations [12]. An example of possible mass median aerodynamic diameter distribution of welding processes after gravimetric measure is reported in Figure 1. Small particles with diameters below 0.05 µm are mostly metal oxide compared to larger particles, which also contain volatile elements (e.g., potassium, fluorine, sodium sulphur). Some studies have shown that almost all Cr and over half of Ni and Mn were in the fraction <300 nm [13].

Since the lung deposition of welding particles depends on particle size and morphology (density, shape), which in turn depends on the welding methods, these factors must be considered when developing protective strategies. Inhaled particles may deposit throughout the respiratory system, but are also exhaled, and different mechanisms affect particle deposition, such as impaction, sedimentation, and diffusion. Generally, particle capability to penetrate deeper into the respiratory system is inversely related to particle size [3,14]. However, the smaller the size, the more complicated become their behavior. In fact, the mechanisms and the efficiency of particle deposition in the respiratory tract depend not only on aerodynamic but also thermodynamic diameter of the inhaled particles. Nanoparticle deposition occurs with high efficiency in the entire respiratory tract due to diffusion, which makes thermodynamic diameter more relevant than aerodynamic diameter because drag forces are absent [15].

The reference standard for particle deposition is the International Commission on Radiological Protection (ICRP) model, where the mechanic deposition (sedimentation, impaction) increases with particle diameter, with a consequent decrease in diffusion, which is particularly relevant for particles <100 nm [16]. In the submicron and micron range (0.1–2.5 µm), the difference between optical diameter and aerodynamic diameter is a decisive factor, since an increase in particle density due to metallic composition makes particles more prone to deposition than their water counterparts [17]. 

The quantification of particle deposition and its clearance in the human airway are essential for evaluating health risks. One of the earlier models to estimate the dose of inhaled particles in the respiratory tract is the U.S. Environmental Protection Agency (EPA) model of the regional deposited dose (RDD) [18]. Use of this model has decreased over time with the development of alternative tools, such as the Multiple Path Particle Dosimetry (MPPD) [19,20], a mechanistic dosimetry model that facilitates the calculation of regional and site-specific deposition and clearance of particles. By adopting the MPPD model, the total deposition, regional deposition, and lobar deposition fraction per airway generation can be quantified. In addition to MPPD, other dosimetry models are available, but may require more expertise in toxicokinetic and computer modeling [21].

**Figure 2:**
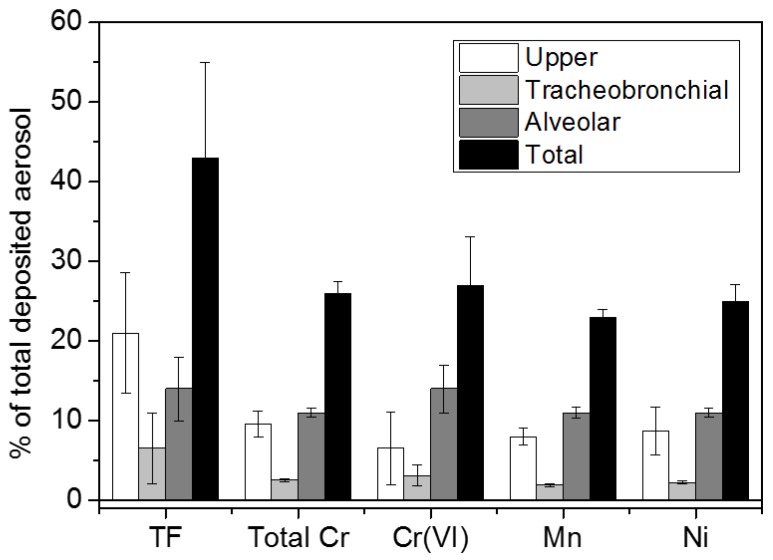
Percentage of total deposited aerosol of metal particles arising from stainless steel welding based on aerodynamic diameter (calculated by particle optical diameter, dynamic shape factor, and density). Adapted by Cena et al [14]. TF: total fume; Cr: chromium; Cr VI: hexavalent chromium; Mn: manganese; Ni: Nickel. For mild steel, percentage of total deposited aerosol are for TF 12, 6.3, 17, and 38 and for Mn 7.8, 2.0, 11, and 23, respectively, in upper, tracheobronchial, alveolar, and total regions.

Recent studies have shown that predicted total respiratory deposition for metal particles was approximately 25% (head airways 7–10% and alveolar region 11–14%), and, of note, Cr VI deposition was highest in the alveolar region [13,14,22]. Furthermore, a significant fraction of the metals may reach the airways in the nanoscale and deposit by diffusion, especially in the case of GTAW [3,11,22]. Figure 2 shows the percentage of total deposited aerosol metal particles arising from SS-WFs based on their aerodynamic diameter [14]. Lung retention time of particles depends on the deposition site and on its interaction with inner lung surface. Retention time is generally short for those particles which deposited in conducting airways (due to mucociliary clearance), but it increases with airway generation number (as a consequence of increasing pathway length and decreasing mucus transport velocity towards distal airways) [23]. There are three major transport pathways for biopersistent particles from the peripheral lungs: (1) alveolar-macrophage (AM) -mediated transport from the alveoli to the ciliated airways, (2) mucociliary transport to the larynx and subsequent swallowing into the gastrointestinal tract, and (3) particle transport towards lung-associated lymph nodes and translocation into blood circulation for subsequent accumulation in secondary organs [24].

Macrophage clearance depends on site of deposition, the amount of particles deposited, and on particle characteristics such as size, shape, and surface reactivity. The predominant mechanism by which insoluble particles are thought to be cleared from the lung is by the cephalad transport of AMs with their phagocytized burdens of particles up the conducting airways [25]. The dust overloading of the lungs is a condition reported in various chronic inhalation studies and reflects a loss of the dust-laden AM mobility [26,27,28,29]. Morrow et al. established that the inability of the dust-laden AMs to translocate to the mucociliary escalator is correlated to an average composite particle volume per AMs in the lung [30]. It appears also that there are significant particle size-dependent differences in the cascade of events leading to effective AM-mediated clearance. In rats, which were exposed to different-sized particles, approximately 80% of nanoparticles (NPs) were retained in the lung, while approximately 20% of the larger particles, >0.5 μm, remained in the lung [31]. The solubility of individual metals varies greatly and depends on welding technique. For instance, those fumes which are generated during MMAW are mainly water soluble, whereas GMAW fumes are relatively insoluble [4]. In this regard, concentration of water soluble Cr depends on welding type and is particularly high in the case of MMAW-SS [32]. The physical characteristics of the fume appreciably affect the kinetics of the clearance of metals from the lungs. In rats treated with intratracheal instillation of MMAW-SS fumes (0.2 mL of suspension composed of 1% of the activated fume in 0.9% sodium chloride), Fe removed from the lungs with a half time of 73 days, and Cr and Ni disappeared with a half time, respectivelyof 53 and 49 days. Instead, rats treated with intratracheal instillation of MIG-SS fumes (at the same dose) showed a different metals’ clearance: No decrease was seen in the pulmonary content of Fe, Cr, and Ni two months after the exposure. By day 103, all metals showed a small decrease [33]. Welding processes with high mass emission rates (MMAW, MAG, MIG, metal inert gas soldering, and laser welding) show mainly agglomerated particles (diameters above 0.1 µm), whereas welding processes with low mass emission rates (GTAW and resistance spot welding) emit predominantly ultrafine particles (diameters below 0.1 µm) [11]. Chang et al. investigated physicochemical and toxicological properties of WFs particles. Fine and ultrafine particle size ranges were found to generate the highest activity in reactive oxygen species and should be evaluated carefully for risk assessment. Spherical particle diameter is inversely related to surface/volume ratio, and increased surface area is accompanied by increased chemical reactivity. Chemical mechanisms include the production of reactive oxygen species (ROS), dissolution and release of toxic ions, disturbance of the electron/ion cell membrane transport activity, oxidative damage through catalysis, lipid peroxidation, or surfactant properties. Despite lung defense mechanisms, such as mucus and mucociliary escalator, NPs seem able to translocate from the lung into the liver, spleen, heart, and possibly other organs with the main mechanism for NPs’ translocation being via endocytosis of alveolar epithelial cells [32]. 

### 3.2. Toxicology Mechanisms of WFs

Toxic and carcinogenic effects of WFs on respiratory diseases were extensively investigated in in vitro, in vivo, and in human studies.

#### 3.2.1. In Vitro Studies

WFs from SS and MS may generate reactive oxygen species (ROS) and ROS-related damage over a range of particle sizes. SS containing Cr and Ni fumes consistently showed a significantly high reactivity, radical generation capacity, and mutagenic activity [34,35]. MMAW-SS fumes showed a toxic, transforming effect on baby hamster kidney cells, which was shown to be Cr VI related [36]. Both soluble and insoluble fractions of MMAW-SS fumes induced sister chromatid exchange. However, mitotic delay could not be explained in terms of Cr VI concentration alone. This indicated that other fume components of both soluble and insoluble fractions were capable of inducing mitotic delay [37]. Previous studies have established that MMAW-SS fumes were more cytotoxic for macrophages and induced a greater release of ROS and secretion of tumor necrosis factor alpha (TNFα) and interleukin-1 beta (IL-1β) compared to GMAW-MS fumes [38,39]. The pulmonary toxicity of complex metal-containing particulates can be associated with soluble forms of transition metals and their doses [40,41]. Soluble fractions of WFs play a fundamental role in mediating proinflammatory responses. Alveolar epithelial cells treated with WFs produced by two Ni-based and one cobalt-based SS welding consumables at concentrations of 31 and of 63 μg ml^−1^ for 6 and 24 h demonstrated a significant increase in IL-8 production at both time points [42]. Treatment with a soluble MMAW-SS sample, at concentrations of 6.25, 25, or 100 μg/mL for 24 h, caused a concentration-dependent increase in DNA damage and lung macrophage death [43]. Mouse macrophages exposed for 24 h to two doses of 50 µg/mL and 250 µg/mL WFs containing Cr VI and Mn were found to be more reactive in terms of ROS production compared to macrophages exposed to the same doses of WFs containing Ni and Cu. However, fumes containing Ni and Cu were able to induce cell death and mitochondrial dysfunction at lower doses. Therefore, reducing the Cr VI and Mn content of generated fumes and increasing the concentration of other metals may not necessarily improve welder safety [44]. In conclusion, SS-WFs containing Ni and Cr are responsible for cytotoxicity, oxidative damage, and mutagenicity effects, while soluble fractions of WFs play a key role in mediating proinflammatory responses. However, both MS-WFs and WFs insoluble fractions may have toxic and mutagenic effects, too.

#### 3.2.2. In Vivo Studies

Several in vivo studies using inhalation, intratracheal instillation, or pharyngeal aspiration of fumes were conducted in order to investigate the course of inflammation and DNA damage due to WFs. Intratracheal instillation is a widely used procedure for delivering materials into the lungs. Reasons for employing this method include its simplicity, relatively low cost, and the ability to deliver a well-defined dose. Because of the nonphysiologic nature of particle delivery, the technique cannot be used to determine lung particle deposition patterns that would occur following inhalation [45]. Pharyngeal bulk particle aspiration from filters can have greater agglomeration with larger diameters, while particles generated during inhalation generally have significantly smaller diameters. These results suggest less reactivity and surface area for aspiration compared to inhalation [46]. Many in vivo studies show that WFs cause inflammation, oxidative and genotoxic damage, and carcinogenic effects. SS fumes were more pneumotoxic and were retained in the lung longer than MS fumes. The MMAW-hard surfacing (HS) fume contains higher levels of soluble Mn and Cr compared to GMAW-MS. MMAW-HS is a welding process used to ensure superficial hardening with wear-resistant material that is applied in specialized settings such as in shipbuilding and railroad industries. Rats treated with intratracheal instillation (0.5 mg/rat, once a week for 7 weeks) of WFs generated by MMAW-HS, rather than GMAW-MS, showed significant lung damage early after treatment and until 35 days [47]. In rats exposed by inhalation to 40 mg/m^3^ for 3 h/day for 3 days to SS-WFs, lung injury and inflammation were significantly elevated at 8 and 21 days compared to controls. By 42 days after exposure, a higher total metal concentration was observed in the lungs of the SS-WFs compared with the MS-WFs, (47% vs. 30%) [48]. After moderate short-term exposure (57–67 mg/m^3^ for 2 h per day in an inhalation chamber for 90 days) of rats to MMA-SS WFs, recovery from pneumoconiosis was observed. A significant dose (105–118 mg/m^3^ for 2 h per day in an inhalation chamber for 90 days) of WFs was required to induce lung fibrosis. Doses were selected based on actual exposure-monitoring data in the shipbuilding industry [49]. Rats exposed to inhalation of MS WFs at a concentration of 40 mg/m^3^ × 3 h/day × 3 or 10 days showed suppressed lung defense responses to bacterial infection as well as rats treated by intratracheal instillation of MMA-SS at concentration of 2 mg/rat. For MMA-SS WFs, soluble Cr was likely the causative agent [50,51]. SS fumes cause inflammation via activation of redox-sensitive nuclear transcription factors, factor kappa B (NF-kB) and activator protein-1 (AP-1), and determine an enhanced release of TNFα and IL-1β from lung cells of rats treated by intratracheal instillation of fumes at a concentration of 1.0 mg/100 g body weight. This dose was chosen after a preliminary dose-response study was performed. When using 0.2 mg, no significant inflammatory responses were observed [52,53]. Respirable-size fumes were collected during GMAW using a SS consumable electrode. Rats were dosed intratracheally with 30 minutes (fresh) WFs and at 1 and 7 days (aged) after fume collection at a dose of 1.0 mg/100 g body weight. Freshly generated SS WFs induced greater lung inflammation than “aged” fumes. This is likely to be due to a higher concentration of ROS on fresh fume surfaces [54]. Several studies have already been performed to evaluate the genotoxic effects of WFs through the use of 8-hydroxydeoxyguanine (8-OH-dG). After 1 day and after 30 days of MMA-SS welding-fume exposure at concentrations of 65.6 +/− 2.9 (low dose) and 116.8 +/− 3.9 mg/m^3^ (high dose) for 2 h per day in an inhalation chamber for 30 days, the amount of 8-OH-dG in lung tissue showed a dose-dependent increase when compared to unexposed controls (73.9 ± 3.4 and 130.6 ± 3.2 vs. 42.5 ± 1.2, respectively) and could reflect oxidative DNA damage to lung cells. The doses were selected based on previously published and actual exposure-monitoring data [55]. A lung tumor susceptible strain (A/J) showed persistent bronchiolar and peribronchiolar change after 1 week of exposure to 40 mg/kg WFs SS by pharyngeal aspiration [56]. After exposure to 5 mg/kg GMAW-MS and GMAW-SS WFs by pharyngeal aspiration four times, strain A/J also showed prolonged dysregulation of immunomodulatory genes when compared to the resistant strain (B6). The cumulative lung fume burden was equivalent to about 196 days of exposure in a 75-kg welder working an 8-h shift [57]. Also GMAW-MS fume, despite containing no metals currently classified as carcinogens, promotes lung tumor in mice. Male A/J mice were exposed by whole-body inhalation to GMAW-MS aerosol for 4 h/day for 4 days/week for 8 weeks at a mean concentration of 34.5 mg/m^3^. Results highlighted an increasing of lung tumor multiplicity and absence of lung inflammation [58]. To identify metal components that are most toxic and tumorigenic, metal components of GMAW fumes were investigated. Lung tumor-susceptible A/J mice were exposed by oropharingeal aspiration to a low or high dose of surrogate metal oxides based on the respective weight percent of each metal in the fume: Cr_2_O_3_ + CaCrO_4_ (366 + 5 µg and 731 + 11 µg), NiO (141 and 281 µg), and Fe_2_O_3_ (1 and 2 mg). Results provided experimental evidence that pneumotoxic effects were negligible for NiO, acute but not persistent for Cr_2_O_3_ + CaCrO_4_, and persistent for the Fe_2_O_3_ exposure, indicating that Fe_2_O_3_ is an important mediator of welding fume toxicity [59].

Evidence for a weak carcinogenic effect in lung tumor-susceptible mice was found for particulate matter. In mice treated with lung tumor initiator 3-methylcholanthrene and exposed to GMAW-SS WFs with 5 weekly pharyngeal aspirations at 340 or 680 μg/exposure, a significant increase in tumor multiplicity was detected in both low- (12.1 ± 1.5 tumors/mouse) and high- (14.0 ± 1.8 tumors/mouse) exposed groups compared to unexposed WFs mice (4.77 ± 0.7 tumors/mouse). Therefore, GMAW-SS particulate matter (PM) acts as a lung tumor promoter in vivo [60]. In tissue samples from both welder case studies and treated rats with SS WFs by intratracheal instillation at a dose of 2 mg once a week for 28 weeks, agglomerates of deposited welding particle compounds by metal complex with Fe, Cr, and Ni were observed within lung cells, particularly AM. A mathematical calculation was used to relate the pulmonary (intratracheal instillation) dosing paradigm employed in this study to workplace exposures of welders [61]. Thus, the pulmonary exposure regimen used in this study mimicked an approximate 10 years’ worker exposure at 200 working days per year [62]. 

Many in vivo studies show that SS-WFs are retained in the lung for a longer period compared to MS-WFs and may cause a higher degree of inflammation, oxidative damage, and genotoxic effects. These effects could mainly be due to soluble Cr, even if Mn, which is contained in MMAW, may play a key role. Low short-term exposure causes reversible effects, compared to high short-term exposure, which can cause irreversible effects such as lung fibrosis. Long exposure to low and moderate doses that are more representative of the working reality can induce lung cancer. In particular, PM both in SS and MS WFs would seem to be a lung tumor promoter.

#### 3.2.3. Human Studies

Chronic exposure to WFs from both SS and MS could lead to oxidative stress and increased levels of chromosomal aberrations and DNA breaks in lymphocytes [62,63]. DNA damage in peripheral blood lymphocytes could be an index of genotoxicity in welding [64]. Genotoxic effects have also been evaluated using the micronucleus test on welder oral mucosa cells, showing an increase in binucleated cell counts and condensate-chromatin cells in welders compared to controls (8 vs. 2 and 4 vs. 0, respectively; values were expressed as number of occurrences per 2000 cells) [65]. Wultsch et al. showed increase of the micronucleus, nuclear buds, and binucleated cells in the nasal cells of welders, with respect to controls (respectively 97%, 63%, and 22%). Moreover, they observed associations between exposure to molybdenum and nuclear abnormalities in buccal cells and between exposure to Ni and nuclear buds both in nasal and buccal cells [66]. Welders exposed to MS-WFs and to SS-WFs experienced an increase in oxidative DNA damage, evaluated by plasma and urinary 8-OHdG. Welders who were exposed to aluminum TIG WFs for 60 minutes showed a 45% increase in urinary 8-OHdG and a 14% increase in plasma 8-OHdG after 3 h, which is a pre-mutagenic DNA adduct implicated in carcinogenesis. An exposure-response relationship was found between particle number concentration in WFs and plasma 8-OHdG concentration [67,68]. Acute and chronic exposures to particulate matter 2.5 (PM_2.5_) generated from welding activities was associated with a modest change in DNA methylation of the inducible nitric oxide synthases (iNOS) gene. Future studies are needed to confirm this association and determine the role changes have in NO production, since NO also plays a role in a variety of cardiopulmonary processes, including asthma, chronic obstructive pulmonary disease, and cardiovascular diseases [69]. Compared to controls, MMAW-SS welders experienced a significant increase in urinary malondialdehyde (MDA) (1.67 ± 1.13 µmoL/g creatinine vs. 0.81 ± 0.26 µmoL/g creatinine) and a decrease in glutathione (GSH) concentration, expressed as GSH/cysteine, in lymphocytes (2.97 ± 0.79 vs. 7.59 ± 3.42). There was also a positive correlation between plasma Cr and urinary MDA, expression of a dose-effect relationship [70]. However, MDA production may also be due to other WFs components such as O_3_ MIG. MIG and TIG welders exposed to ozone concentrations of 0.30 ± 0.19 parts per million (ppm) presented serum MDA levels higher than the controls and a significant correlation between them was detected. Duration of exposure, which appears to be responsible for 54% of changes in serum MDA levels, is also important [71]. Occupational exposure to WFs may also induce a transient acute inflammation, as shown by increased values of endothelin-1 and neutrophils in the blood of MMA welders with respect to MAG welders directly and after 24 h of exposure to WFs (2.5 mg/m^3^ for 6 h/day for 3 days). No differences in inflammation indices after 1 and 7 days of exposure was detected [72]. PM_2.5_ concentrations (median: 1.66 mg/m^3^) in apprentice welders were found to be significantly associated with absolute neutrophil counts in nonsmokers and C-reactive protein levels in both nonsmokers and smokers [73]. In a population of patients, 21 former welders and 21 controls, underwent surgical resection for nonsmall-cell lung cancer and fibrotic regions in nonneoplastic lung tissue samples were more severe in welders than to controls. NPs (Fe, Mn, Cr oxides, essentially) were found in macrophages and in lung fibrotic regions of welders. In vitro, it was demonstrated that exposure of macrophages to Cr and Mn oxides NPs may increase the production of motif chemokine ligand 8 (CXCL-8), IL-1β, TNFα, C-C motif chemokine ligand 2(CCL-2), -3, and -4, and to a lesser extent IL-6, and CCL-7 and -22 [74]. Most WFs metals were in the fraction of NPs. Compared to larger particles, NPs have been associated with greater ROS activity [22]. Finally, WFs could decrease alveolar clearance, since there was a negative correlation between clearance half-time value and forced expiratory volume in 1 second (FEV_1_) and forced vital capacity (FVC) [75]. In conclusion, MS and SS WFs are clearly pneumotoxic substances and the vast majority of WFs metals were found in NPs. Lung particle deposition is particularly important for NPs compared to larger particles, since NPs cause a greater release of ROS, resulting in target organ damage. Moreover, a relationship between exposure to NPs and long-term lung effects in welders was observed.

When welders are exposed to a high concentration of hazardous substances in WFs over a longer period, then this may lead to a strain on the respiratory and cardiovascular systems. Health effects due to WFs exposure can be seen, therefore, as an endpoint of repeated acute damages that lead to a progressive remodeling and fibrosis. In this context, biomarkers could be used to assess the harmful potential of WFs exposure not only as an indirect index of dose, but also to quantify the risk of adverse effects in susceptible workers. Furthermore, the periodic search of early biomarkers of lung and cardiovascular effects will probably overcome the limitations of traditional imaging or functional procedures, which often indicate late and frequently irreversible dysfunctions.

### 3.3. Lung Disease

Respiratory diseases may occur in welders. These include benign and reversible diseases: metal fume fever, siderosis, and pulmonary function abnormalities; acute diseases: infectious pneumonia; chronic diseases: fibrosis, asthma, chronic bronchitis, and chronic obstructive pulmonary disease; and malignant diseases: lung cancer. 

#### 3.3.1. Metal Fume Fever

Metal fume fever (MFF) is an acute self-limited respiratory illness that occurs approximately 4 h after exposure and usually resolves in 24 to 48 h after onset. It is a flu-like syndrome caused by inhalation of WFs containing Zn but also Cu, Mg, and Cd. The incidence in the US is 500–2000 cases/year and 30% of welders experience one or more episodes [76]. Pulmonary responses to inflammatory cells could play a large role in MFF as shown by dose-dependent increase in the number of polymorphonuclear (PMN) recovered in bronchoalveolar lavage (BAL) fluid 22 h after exposure. It has been also demonstrated that exposure to Zn fumes causes ROS formation in human PMN. TNFα was a key mediator in MFF initial response while IL-6 and IL-8 increase was involved in later response [77,78]. MFF could be a predictor for the development of respiratory symptoms suggestive of welding-related asthma. Apprentice welders with possible MFF had an increased risk of developing these symptoms, but not pulmonary functional abnormalities [79]. In a controlled human study, volunteers exposed at 5 mg/m^3^ of Zn oxide fume produced fever and symptoms such as fatigue, muscle ache, cough, and an increase of IL-6 plasma levels. After exposure at 2.5 mg/m^3^ subjects produced fever and increase of IL-6 plasma levels in absence of symptoms [80]. IL-6 showed a significant increase also in subjects exposed to WFs containing Zn at concentration of 1.5 mg/m^3^ and Cu at concentration of 0.4 mg/m^3^. It was seen that IL-6, an acute phase mediator, increased 6 to 10 h after exposure and, therefore, could represent an early biomarker of exposure to WFs containing Zn and Cu [81].

In summary, MFF is a self-limiting illness and one of several febrile flu-like respiratory syndromes encountered in the occupational setting. It is the classic example of inhalation fever, but its presence must not be unreported. In fact, an episode of MFF may identify a worker who may be at risk for developing other welding-related lung diseases, such as asthma.

#### 3.3.2. Infectious Pneumonia

**Figure 3:**
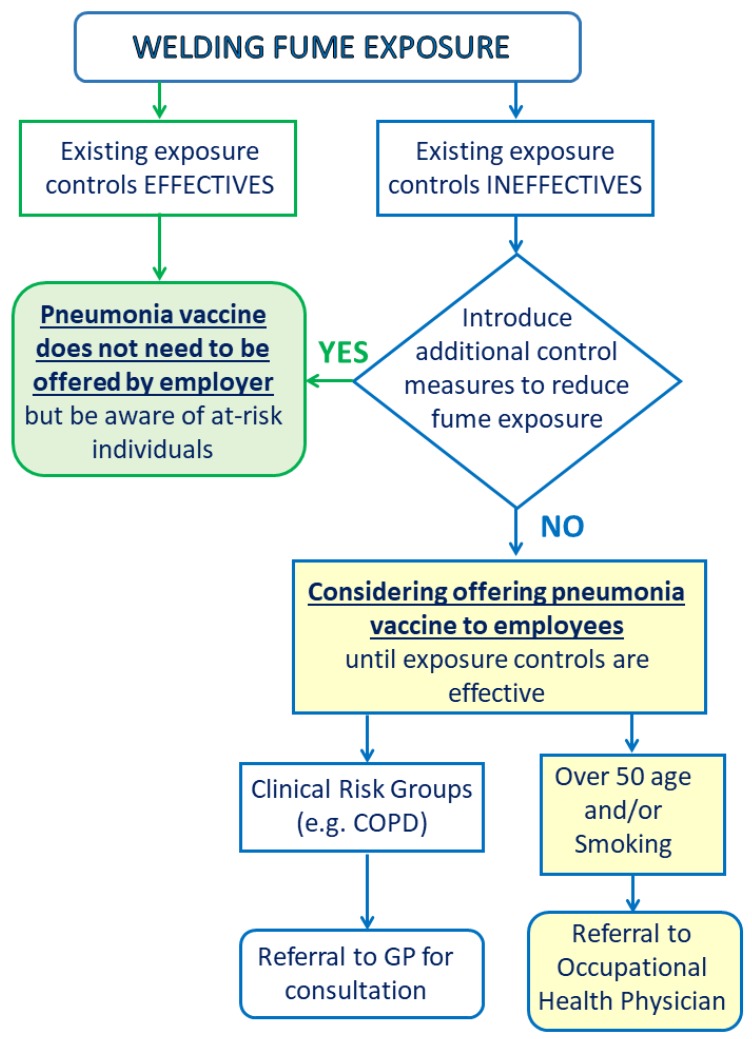
Simple pneumonia vaccine decision scheme. COPD: chronic obstructive pulmonary disease; GP: general practitioner.

Welders have an increased risk of developing pneumococcal pneumonia, a well-known fact since the 1950s. An increased pneumonia risk is a constant threat throughout a welder’s working life [82], and further analyses of occupational mortality in the UK over more than five decades have demonstrated increased pneumonia death rates in welders. However, no excess mortality has occurred in men above retirement age [83]. In a case control study, pneumonia was associated with occupational exposure to metal fumes the previous year but not in earlier periods and the risk was highest for lobar pneumonia and recent exposure to ferrous fumes [84]. To confirm its continuing relevance in more recent working conditions, analysis of occupational mortality for England and Wales was extended to the period 1991–2000. Among men of working age, excesses of pneumococcal pneumonia were noteworthy in welders and in sheet metal workers (proportional mortality ratio (PMR) = 268, 95% confidence intervals CI: 143, 459). However, no excess was found from these causes among older age groups or from bronchopneumonia at any age [85]. A recent prospective (from 1971 to 2003) cohort study involved a study population of 320,143 male construction workers, of whom 79,305 were unexposed (controls). Findings showed an increased mortality from infectious pneumonia among construction workers exposed to metal fumes, inorganic dust, and chemicals [86]. As for the pathogenic mechanism, Streptococcus Pneumoniae adherence to lower airway cells is facilitated by an interaction between bacterial phosphorylcholine and the platelet-activating factor receptor (PAFR) expressed on host cells [87]. MS WFs increase PAFR-dependent pneumococcal adhesion and infection, and SS WFs stimulate lung PAFR mRNA expression and PAFR-dependent pneumococcal adhesion and infection to lower airway cells. In this study, the airway cells were exposed at WFs concentrations 105 to 145 µg/cm^2^ for 2 h. Furthermore, it was shown that exposure at lower concentrations for longer duration (5 µg/cm^2^ for 24 h) induced also pneumococcal adhesion and infection [88]. In 2011, the pneumococcal polysaccharide vaccine (PPV23) was recommended by the Department of Health (DH) in UK for welders. The vaccine is safe and antibody response to a single dose of PPV23 is developed after three weeks. There is current uncertainty regarding optimum vaccination timing. An argument can be advanced for only offering vaccination to welders from older age groups (e.g., over 50 years) since the incidence of invasive pneumococcal disease in the general population rises with age [89]. Subsequently, the DH relaxed its recommendations on the advice of the UK Health and Safety Executive (HSE). HSE guidelines emphasized that vaccination was not a regulatory requirement, provided that an adequate risk assessment could demonstrate that fumes had effectively been controlled [90]. Figure 3 represents a possible pneumonia vaccine decision scheme. Furthermore, despite a reduction in exposure levels, mortality from pneumococcal and unspecified lobar pneumonia during 2001–2010 increased significantly in welders of working age with a particularly high proportional mortality ratio (PRM) for pneumococcal pneumonia between 2001–2010. Authors claim that a considerable proportion of the disease could have been preventable with the pneumococcal vaccination [91].

Inhalation of fine and ultrafine particles, including those in WFs, may affect pulmonary immunologic defenses. Severe and sometimes fatal cases of Bacillus Cereus (BC) pneumonia in apparently healthy welders have been reported [26,92]. In these cases, a strain of BC that possesses the anthrax toxin genes and is capable of causing a severe inhalation anthrax-like illness has been implicated. Different strains of BC are responsible for pneumonia in welders. It was demonstrated that the genome sequence of BC G9241, isolated from a welder in Louisiana, harbors almost the entire pXO1 anthracis virulence plasmid [92]. BC 03BB102 and 03BB108, isolated from another welder in Texas, did not contain genomic sequences of sufficient homology, yet the capA, capC, and capB genes, which are required for biosynthesis of the B. anthracis capsule, were detected in both genome sequences [93]. These results demonstrate the need for increased awareness of occupational risk factors that may increase the susceptibility of welders to infection by B. cereus. In addition, authors reported that WFs reduced the cytotoxic activity of lymphokine-activated killer cells which are primarily natural killer (NK) cells. They suggested that the decreased immunocompetent state might subject the welders to an increased risk of infection because NK cells are a main defense mechanism against viruses [94].

Unfortunately, many employers (and welders) are unaware that exposure to WFs can cause pneumonia. In view of this limitation, there is a need for proper risk information and data collection. In fact, a better understanding of the mechanisms is needed because the rarity of lobar pneumonia makes it difficult to establish a correlation through epidemiological studies. What is more, an improvement in the knowledge of infective mechanism will lead to a better identification of those workers more susceptible to work-related infections.

#### 3.3.3. Pulmonary Function Abnormalities

Pulmonary function studies (Table 2 and Table 3) have shown contrasting results due to many confounding variables (welding processes and materials used, duration of exposure, exposure area ventilation, effects of population dynamics, effects of smoking) [4]. In many of these studies, the annual decline of FEV_1_ was greater in welders than in controls, mainly in smoking welders [95,96,97,98,99]. Use of MMAW (that generates greater quantities of fumes in comparison to other processes) and occupational exposure in confined spaces (e.g., shipyard welders) would seem to be associated with lung function abnormalities [95,100]. Furthermore, a significant decline of FEV_1_ and FVC was observed in some studies and appears to be associated with occupational exposure duration [98,101,102,103]. In a meta-analysis that included five case-control studies, the pooled estimate of the difference in FEV_1_ decline between welders and nonwelders was −9.0 mL/year. After adjustment for smoking status, in smokers the pooled estimate of the difference in FEV_1_ decline among welders and non-welders was −13.7 mL/year and −3.8 mL/year, respectively [104]. Stopping smoking, adequate ventilation systems, and correct use of collective and individual devices are all safety procedures to be implemented for risk reduction strategies.

#### 3.3.4. Chronic Bronchitis

Chronic bronchitis (CB) is defined as cough and sputum production for at least 3 months in each of two consecutive years [105]. Occupational exposure to WFs has been associated with an increase in frequency of CB symptoms [102,106,107]. There is an interaction between smoking, WFs exposure, and CB. A recent study which included a sample of 15,909 subjects from the general population in Northern Europe showed that smoking and occupational exposure to WFs are both associated with an increased prevalence and incidence of CB [106]. A pathogenetic study evaluated oxidant-antioxidant status in 34 welders and 20 controls. Serum levels of oxidant agents, thiobarbituric acid reactive substances (TBARS) and protein carbonyls were higher in welding workers than those in controls, whereas serum levels of antioxidant agents, erythrocyte-reduced GSH, and total protein thiol (SH) group were significantly lower in welders than in control subjects. Respiratory symptoms were also found to be significantly more prevalent in cases than in controls, suggesting possible impaired oxidative-antioxidative balance effects in CB pathogenesis [108].

#### 3.3.5. Asthma 

Occupational asthma (OA) is a disease characterised by variable airflow limitation and/or hyperresponsiveness associated with inflammation due to causes and conditions attributable to a particular occupational environment and not to stimuli encountered outside the workplace [113]. OA may develop as a consequence of exposure to some metals present in WFs, which may induce airway sensitization [2], but WFs may also cause irritant-induced asthma [114]. However, a recently published large population-based study based on a 9-year follow-up of the first European Community Respiratory Health Survey (ECRHS) did not show any overall association between welding and asthma [107]. In contrast, the Shield surveillance program showed that welders accounted for 9% of reported OA cases [115]. The likely incidence of OA in a population study of 194 apprentice welders newly exposed to WFs was 3% (6 out of 194) and the incidence of bronchial hyperresponsiveness was 11.9%. The authors concluded that exposure to WFs and gases, even for a brief period, is associated with pulmonary functional changes and respiratory symptoms [116]. A recent Danish study indicated that long-term exposure to high level (>100 mg/m^3^-years) of SS WFs compared to low levels of exposure (<15 mg/m^3^-years) was related to an increase in an asthma hazard ratio (HR) in nonsmokers [(1.46, 95% confidence interval (CI): 1.06–2.02)] [117]. The mechanism through which WFs exposure may induce OA is not fully understood. In some studies, an Immunoglobulin E (IgE)-mediated mechanism was suggested, although skin reactivity to metal salts was negative. A Finnish clinical study, which included 34 SS welders with OA, investigated the mechanism associated with OA related to SS welding and concluded that, in addition to a possible IgE-mediated mechanism, other immunological mechanisms may also exist. Interestingly, after OA diagnosis, the continuation of welding work was possible only in 6 out of 31 welders [118]. Several case reports have revealed OA in welders, especially in SS welding [119]. The onset of respiratory symptoms may lead to diagnosis of asthma immediately or later, up to 8 years [119,120,121]. The first case of Mn-induced OA in a welder was published in 2008; this worker developed a 55% decrease in FEV_1_ and dyspnea and an increase in eosinophils count in sputum after a bronchial challenge with 0.1% Mn chloride solution [122]. In conclusion, although the first ECRHS study did not reveal any overall association between welding and asthma, OA might occur in welders. Cr and Ni are two known sensitizers, while other metals, such as Mn and other WFs agents, may induce OA.

In conclusion, although the first ECRHS study did not reveal any overall association between welding and asthma, OA might occur in welders. Cr and Ni are two known sensitizers, while other metals, such as Mn and other WFs agents, may induce OA.

#### 3.3.6. Chronic Obstructive Pulmonary Disease

Chronic Obstructive Pulmonary Disease (COPD) is a common, preventable, and treatable disease that is characterized by persistent respiratory symptoms and airflow limitation due to airway and/or alveolar abnormalities usually caused by significant exposure to noxious particles or gases [105]. Cigarette smoking is undoubtedly the main cause of COPD in the general population, but harmful workplace exposure has been linked to an increased prevalence of COPD [123]. Interest in COPD in nonsmokers has increased in the past decade, a growing number of published studies have suggested that risk factors other than smoking are strongly associated with COPD [124]. Prevalence of COPD in 12,980 never-smoker participants in one of the three US-based National Health and Nutrition Examination Surveys (NHANES) was 5.1% [125]. Ten years later, the NHANES III study reported that the prevalence of COPD in never-smokers was 6.6% [126]. The NHANES III survey data were analyzed in an advanced epidemiologic study conducted from 1988 to 1994 on a population of 9823 subjects aged 30–75 years; it estimated the fraction of COPD attributable to work as 19.2% overall and 31.1% among never-smokers [127]. Occupational and environmental factors causing COPD include organic and inorganic dusts, metal fumes, chemicals, and gases [128]. A systematic review of scientific literature has shown a significant association between occupational exposures and COPD in 22 out of 25 population-based studies, 12 out of 15 studies with inorganic/mineral dust exposure, and 17 out of 19 studies on organic exposure [129]. A case-control study (131 cases of COPD and 298 controls) was conducted to ascertain COPD risk comparing different occupations to office workers: welders showed age- and smoking-adjusted odds ratio (OR) for COPD to be 6.4 and presented the highest increase in COPD risk for each extra year of exposure [130]. A potential relationship between WFs exposure and COPD has been suggested in 240 welders working in two shipyards in Korea and a significant excess risk was found in the intermediate (OR = 3.9, 95% CI: 1.4–13.3) and high (OR = 3.8, 95% CI: 1.0–16.2) exposure group compared with the low exposure group. Cumulative WFs exposure in low, intermediate, and high exposure groups was, respectively, of 0.1–3.4, 3.4–11.7, and 11.7–22.8 mg/m^3^-years [131]. Interestingly, a relationship between welding fume exposures and COPD has not always been demonstrated. For instance, in the WELDOX German study, authors showed that that age- and smoking-adjusted lung function parameters showed no decline with increasing duration, current exposure level, and lifetime exposure to WFs. Furthermore, despite the fact that 15% of the welders had FEV_1_/FVC below the lower limit of normal, the authors could not substantiate the presence of an association with the measures of exposure [132]. Furthermore, Christensen et al. showed that FEV_1_ values declined, on average, 856 mL in welders at the start of the 18-year follow-up and 704 ml among smoking nonwelders [96]. This larger decline among the welders, amounting to some 150 mL, did not reach statistical significance and differences among welding and not welding nonsmokers were even smaller. In addition, when FEV_1_/FVC ratio was used as outcome, no association with WFs exposure was demonstrated.

#### 3.3.7. Interstitial Lung Disease and Pulmonary Fibrosis

Inhalation of fumes from iron (Fe) and steel has long been known to cause siderosis, which is a benign pneumoconiosis caused by inhalation of particles containing Fe oxides. Nevertheless, apart from a few case reports, most pulmonary fibrosis cases have causes that are not Fe exposure-related. Twelve case series and 34 case reports confirm that the inhalation of WFs for a prolonged period may cause a form of pulmonary fibrosis similar to that presented during respiratory bronchiolitis (RB) and desquamative interstitial pneumonia (DIP) [133]. DIP and RB are interstitial smoking-related pneumonias, but occupational etiology can also be recognized [134]. Although rare, DIP has been described in both Al and steel welders. In symptomatic welders, it would be appropriate to perform further investigations, such as lung plethysmography and high-resolution computerized tomography (HRCT).

#### 3.3.8. Lung Cancer

Cr VI and Ni are classified by the International Agency for Research on Cancer (IARC) as carcinogenic for humans (group 1) and may induce lung cancer [135,136,137]. In 1989, the IARC classified WFs as a possible human carcinogen (group 2B) [138]. In a recent evaluation the working group of IARC concluded that there is “sufficient evidence in humans” that WFs cause lung cancer (group 1) [139]. Pesch et al. [140] investigated the risk of lung cancer after exposure to WFs, Cr (VI), and Ni, analyzing 3418 lung cancer cases and 3488 controls among men from two German case-control studies (1988-1986). ORs for lung cancer with high exposure were 1.55 (95% CI: 1.17; median: 2.05; 1.8 µg/m^3^ × years) for WFs, 1.85 (95% CI: 1.35; median: 2.54; 1.4 µg/m^3^ × years) for Cr (VI), and 1.60 (95% CI: 1.21; median: 2.12; 9 µg/m^3^ × years) for Ni, showing that these three agents might contribute independently to the excess lung cancer risk. Even if their interactions are difficult to interpret, findings support the classification of WFs as carcinogenic to humans. Studies have been carried out and have also shown excess lung cancer risk for MS welders. It has been shown that welding is associated with a 25–40% increase in lung cancer risk, even if the agents responsible for such a high risk have not yet been identified [141,142,143,144]. In a multicenter cohort study of 11,092 welders from 135 companies located in nine European countries, an excess for mortality from lung cancer (116 cases vs. 86.81 expected, standardized mortality ratio (SMR) = 1.34) was observed both in SS and MS welders, although it was greater in SS welders [142]. In a cohort of 10,059 metal workers, the incidence of lung cancer was increased among welders and also for MS welders, where exposure to Cr VI and Ni was negligible [145]. In the occurrence for neoplastic disease, exposure duration seems to be also important. A mortality study conducted on 2721 welders showed an excess risk for lung cancer in MS welders employed for 20 or more years (5 cases vs. 1.54 expected, SMR = 3.24) [146]. In a cohort of 3247 male workers, the mortality risk increase for lung cancer was 32% in welders with respect to the general population, increasing until 74% after 20 or more years of exposure [147]. A subsequent study on a cohort of workers of a shipyard detected a relative risk of 3 for lung cancer only in welders employed more than five years. Environmental monitoring revealed total dust concentration median in WFs of 2.5 mg/m^3^, Cr concentrations below 0.05 mg/m^3^, and Ni concentrations of 0.01 to 0.04 mg/m^3^ [148]. In contrast, in Steendal’s study, no increased risk of lung cancer with increased duration of exposure for MS welders was found. In this study, we recruited welders with no exposure to asbestos, Cr, and Ni. Environmental sampling revealed in these plants total particulate concentrations of 6–7 mg/m^3^. The standardized mortality ratio (SMR) for lung cancer, calculated in a follow-up period of 10 years, was 1.46 for MS welders [149]. Langard et al., after reviewing epidemiological studies, concluded that SS welders have a higher risk of lung cancer than MS welders using the same welding processes. However, it was not possible to clarify whether Ni and Cr VI in SS WFs are the main risk factor for lung cancer [150]. A Danish cohort study showed an exposure-response relationship in SS welders, but not in MS welders. The estimate particulate exposure levels was of 1.6 mg/m^3^ for SS MMA welders, 0.6 mg/m^3^ for SS TIG and MAG welders, 2.7 mg/m^3^ for MS MMA welders, and 2.6 for MS TIG and MAG welders [151]. A successful meta-analysis (1954–2004) including US and European studies showed a 26% excess of lung cancer risk in welders with no difference between MS and SS welders [141]. In partial contrast, studies conducted in Germany and in Norway in the 1990s showed an excess of cancer mortality for mesothelioma, but not for lung cancer [152,153].

The presence of confounding factors should not be underestimated. The shipyard welders may be exposed to asbestos that can cause also lung cancer. Smoking is another causal agent of lung cancer. In a groups of MS MMA shipyard welders, employed between 1940 and 1979, there was found an excess of lung cancer, 9 cases vs. 3.6 expected, standardized incidence ratio (SIR) = 2.50. In this study, exposure to smoking and asbestos were confounding factors [154]. In a meta-analysis of epidemiologic studies, a 30–40% increase in relative risk (RR) for lung cancer was observed in welders and this increase could not be linked to Cr VI and Ni exposure. Confounding factors such as asbestos and tobacco smoke might contribute to high RR [143]. Honaryar et al. performed a meta-analysis of case-control and cohort studies on welding or exposure to WFs and risk of lung cancer, accounting for confounding factors were exposure to asbestos and tobacco smoking [155]. Results showed increased risk of lung cancer, regardless of the type of steel welded, the welding method (arc vs. gas welding), and independent of exposure to asbestos or tobacco smoking. According to other studies, excess cancer risks are still present after adjustment for smoking and in those welders with minimal asbestos exposure [144,156,157]. In an extensive case-control study after adjustment for smoking and asbestos, the dose-response relation was strong in welders with more than 25 years of exposure to WFs, an increase of risk was found. A case-control study performed in Montreal showed a significant increase of risk for lung cancer both in gas (OR = 2.9) and arc (OR = 2.3) WFs in non/low smokers but not in moderate/heavy smokers. The authors concluded that results in heavy smokers might be due to the strong effect of tobacco smoking, which could mask the weak effect of WFs on lung cancer [158]. A recent prospective study revealed a positive relation between duration of exposure in years and lung cancer risk among heavy smokers. The associations were predominantly from increased risk of squamous cell carcinoma (SqCC) [159]. In the SYNERGY study, a link has been suggested between WFs for SqCC and small cell lung cancer (SCLC) [160], confirming previous studies where welders had a high risk of SqCC [158,161,162]. Ambivalent results have been reported on the association between occasional welding exposure and lung cancer risk [163]. A recent cohort study on 12,845 welders, who were followed from 1991 to 2010, showed an elevated hazard ratio (HR = 1.16) for lung cancer and in particular for SCLC (HR = 1.54) and SqCC (HR = 1.19) [164].

Cohort and case-control studies indicate an excess of risk for lung cancer both in SS and MS welders. Some studies have reported a higher risk in SS with respect to MS welders and an exposure-response relationship only in SS welders. However, studies conducted on MS welders alone showed an excess of risk also in these workers. Although tobacco smoke and asbestos are confounder factors, studies of welders not exposed to asbestos and after adjustment for cigarette smoking confirmed the association between exposure to WFs and lung cancer. Further studies are needed to understand if specific components of the welding fumes or if the welding fumes themselves are responsible for the carcinogenicity. Cohort studies and largest case-control studies related to risk of lung cancer in welders are summarized in Table 4 and Table 5.

Lung cancer risks in welders were observable at very low exposure levels. As a result of this IARC evaluation, there is a strong need to review current health-based exposure limits for metal dust and fumes from welding to ensure they are protective.

### 3.4. Occupational Exposure Limits

WFs, Cr VI, and Ni are classified by IARC as carcinogenic for humans (group 1) [138,139]. The American Conference of Government Industrial Hygienists (ACGIH) has set a TLV-TWA (Threshold Limit Value-Time-Weighted Average) of 5 mg/m^3^ for WFs, measured as total particulate in the welder’s breathing zone [165]. As for Cr VI, the National Institute for Occupational Safety and Health (NIOSH) recommends that airborne exposure to all Cr VI compounds be limited to a concentration of 0.2 µg/m^3^ for an 8-h TWA exposure, during a 40-h work week [166]. TLV-TWA set by the ACGIH is 50 µg/m^3^ for Cr VI water-soluble compounds and 10 µg/m^3^ for Cr VI insoluble compounds [167]. The Occupational Safety and Health Administration (OSHA) has recently set the following industrial exposure limits: 2.5 µg/m^3^ as action level and 5 µg/m^3^ as permissible exposure limit [168]. The Scientific Committee on Occupational Exposure Limits (SCOEL) proposes as a limit of exposure 0.025 mg/m^3^ for Cr VI compounds [169]. Occupational limits of exposure recommended by NIOSH for Ni is 0.015 mg/m^3^ averaged over a work shift of up to 10 h per day and 40 h per week [170], while ACGIH proposed a TLV-TWA of 1.5 mg/m^3^ for Ni metal, 0.2 mg/m^3^ for Ni insoluble compounds, and 0.1 mg/m^3^ for Ni soluble compounds [167]. OSHA established a PEL of 1 mg/m^3^ for all Ni compounds [171]. SCOEL recommends two different occupational exposure limits: 0.05 mg/m^3^ for respirable fraction and 0.01 mg/m^3^ for inhalable fraction [172].

However, studies have shown that, in MS welders, respiratory symptoms clearly increase during working days, although the exposure was not extreme compared to the current exposure limit [173]. More recently, Monse et al. [174] reported a concentration-response relationship with nano-sized ZnO particles in a low concentration range was, with systemic inflammatory effects of inhaled nano-sized ZnO particles, observed at concentrations well below the occupational exposure limit for ZnO. These observations clearly indicate that the current occupational exposure limit (OEL) may not be sufficient to fully prevent harm in welders. Therefore, ulterior studies seem necessary to define such dose-response relationships with the aim also to identify adequate limit values for occupational exposure.

### 3.5. Biological Monitoring

Biological monitoring of welders has traditionally relied on urinary and/or blood concentration and urinary Cr measurements are usually used as an indirect monitoring of systemic exposure to ambient Cr VI in FCAW welders, although with limitations [175,176]. SS and MS welders who were exposed to low levels of Cr and steel welders who were mildly exposed had significantly increased levels of Cr in post-shift urine compared with referents (median [0.30 µg/g creatinine] range [<0.1–3.7] vs. <0.1 µg/g creatinine [<0.1–0.22]) [177,178]. Changes in urinary excretion of total Cr from pre- to post-shift can be used to evaluate recent exposure, whereas total Cr in pre-shift urine, blood plasma, and blood erythrocytes reflect long-term exposure to Cr [178]. In urine, Cr is totally reduced to trivalent Cr and it is not possible to quantify the real exposure to Cr VI; therefore, the content of Cr VI in erythrocytes may be used [179,180], since it reflects Cr VI compounds which have entered membrane cells. Exhaled breath condensate (EBC) may be a suitable matrix for assessing lung tissue dose and consequent pulmonary effects [181]. In EBC it is possible to determine biomarkers of exposure, such as metals and particulates, and biomarkers of effects (inflammation and oxidative stress). Unlike urine, the fraction of Cr VI and causes of toxic and carcinogenic effects can be estimated in EBC [182]. About the Mn, blood and urine as matrices of biological monitoring were largely studied; however, Mn levels in blood and urine often were not related to Mn in air [183,184]. Also in this case, EBC could be a valid alternative. In 17 MIG welders, EBC Ni and Mn levels were significantly higher compared to controls (1.00 [0.84–1.46] µg/l vs. 0.24 [0.10–0.65], 4.72 [2.99–17.13] vs. 0.32 [0.16–2.47]), whereas in urine these differences were only present for Ni (1.56 [1.01–2.48] μg/g creatinine vs. 0.95 [0.56–1.59] μg/g creatinine). It appears that Mn-EBC, as well as Ni-EBC, represents a promising marker of exposure to WFs [185]. It was hypothesized that WFs exposure would be associated with pulmonary inflammation as reflected by acute changes in EBC pH values shown in some studies. However, changes in EBC pH should be related to other biomarkers of exposure and effects on EBC [186,187]. Higher concentrations of 8-isoprostane (oxidative biomarkers) and a less acidic pH were detected in EBC of welders using FCAW compared with EBC of welders using GMAW technique [188]. H_2_O_2_ was significantly higher in two groups of welders exposed to Al/Fe and to Cd/Cr/Fe/Pb/Ni compared to controls (0.19 ± 0.11 and 0.19 ± 0.16 µM vs. 0.03 ± 0.04 µM) [187]. Nitrate and EBC markers of nitrosative stress were significantly higher in welders after 3, 6, and 24 h of work [189], with lower levels in the case of personal protection equipment use. Markers of oxidative stress in the condensate are intended to identify early abnormalities in asymptomatic welders without clinical and functional abnormalities and, therefore, may be used for biological monitoring [190]. The effect of MAG fumes was investigated in 12 healthy male subjects showing no changes in inflammatory (nitrate, nitrite, and nitrotyrosine) and oxidative EBC biomarkers (malondialdehyde) [191]. In other studies, higher 8-isoprostane and leukotriene B4 (biomarkers of inflammation) concentrations were revealed in gas arc welders with undetectable Cr in EBC and high Fe and Ni concentrations compared to welders with detectable Cr and low levels of both Fe and Ni in EBC (443.3 pg/mL vs. 247.2 pg/mL, 30.5 pg/mL vs. 17.3 pg/mL). These results suggest irritative effects in the airways of healthy welders [188,192]. In SS welders, Ni and Cr urinary are used as biomarkers of exposure, and Cr in erythrocytes can be used for exposure to fraction of Cr VI. In EBC it is possible to dose metals and biomarkers of effects (inflammation and oxidative stress). Cr, Cr VI, Ni, and Mn in EBC seem to be reliable markers of exposure [181,182,185]. Fe–EBC, on the other hand, does not appear to be a reliable marker of exposure despite relatively high Fe concentrations in WFs [185]. Indicators of exposure and indicators of early effects in welders are generally not correlated with lung function parameters; this is probably because most of the studies have been performed in relatively healthy welders, with largely normal lung function data. For this reason, we speculate that assessing lung oxidative stress and inflammation in welders could be a sensitive endpoint for evaluating early biochemical changes in the airways. This approach will probably overcome the limitations of traditional spirometric tests, which often indicate irreversible pulmonary dysfunction. The use of biomarkers of effect in welder surveillance perspective is a goal that should be reached. However, further studies are necessary to assess whether these biomarkers might be used to identify welders at high risk for developing a respiratory disease since they cannot yet replace blood and urinary biomarkers.

## 4. Discussion

A dose-dependent increase of number of polymorphonuclear (PMN) and cytokines in BAL has been demonstrated in MFF. IL-6 plasma levels increase already after exposure to 1.4 mg/m^3^ zinc oxide fume, and fever and symptoms appear, respectively, after zinc oxide fumes’ exposure at 2.5 mg/m^3^ and 5 mg/m^3^. An increased pneumonia risk is a constant threat throughout a welder’s working life. Rats treated of 40 mg/m^3^ × 3 h/day × 3 or 10 days by inhalation showed suppressed lung defense responses to bacterial infection. Excesses of pneumococcal pneumonia are noteworthy in welders. In vitro studies found that airway cells exposed to MS WFs at concentrations of 105 to 145 µg/cm^2^ for 2 h and at concentration 5 µg/cm^2^ for 24 h showed an increase of PAFR-dependent pneumococcal adhesion and infection. Lung function studies on welders often lack quantification of WFs exposure. In many of these studies, the annual decline of FEV_1_ was greater in welders than in controls, mainly in smoking welders, and appears to be associated with occupational exposure duration. COPD risk also increases with increasing of dose and exposure duration. A cumulative WFs exposure of 3.4–11.7 and 11.7–22.8 mg/m^3^-years compared to cumulative exposure of 0.1–3.4 mg/m^3^-years causes an excess of COPD in welders. OA may occur in welders and diagnosis can be immediate or later, up to 8 years. A higher risk of OA appears in cases of high-level cumulative exposure (>100 mg/m^3^-years) compared to low-level exposure (<15 mg/m^3^-years). After diagnosis of OA, only a smart number of welders can restart welding activity. WFs are carcinogenic for humans (group 1, IARC). Numerous cohort studies, meta-analysis, and large recent case control studies have shown an excess lung cancer risk both in MS and SS WFs. This excess of risk also after adjustment for asbestos and cigarette smoking has been confirmed. Some studies detected an exposure-response relationship, but only for SS WFs. Duration of exposure must be also considered. Lung cancer risk increased, above all, following exposure of >20 years. In vivo studies showed that long exposures to low and moderate doses were responsible for lung cancer. Further studies are needed to realize the exposure-dose, exposure-effect, and dose-effect relationships to the WFs-related respiratory diseases. Furthermore, WFs components responsible for carcinogenicity should be determined. In order to achieve these goals, exposure should be better quantified and characterized.

Early detection of those lung diseases associated with welding exposure is crucial for secondary prevention strategies, since complete avoidance or reduction of relevant exposure is not always possible. Furthermore, a specific approach could be designed for welders, such as pneumococcal vaccination. However, routine reimmunization with PPV23 is not widely recommended because of concerns associated with increased reactogenicity and immunological hyporesponsiveness after repeated dosing [193]. This issue could be overcome by offering welders a conjugate vaccine (Penumococcal Conjugate vaccine (PCV) 13), which has been in use since 2009. PCV13 gives prolonged protection and is responsible for immunological memory and booster effect [194]. The advisory Committee on Immunization Practices recommends the use of a single dose of PCV13 followed by a dose of pneumococcal polysaccharide vaccine 23 (PPSV23) 6–12 months later. Adults who have already received PPSV23 should receive a dose of PCV13 ≥ 1 year after receipt of PPSV23 [195]. However, employers should first prioritize the control of exposure to welding and metal fumes by adopting the hierarchy of control measures specified under the local regulations before considering offering the pneumonia vaccine to their employees. If fume exposure is well controlled, then it is up to individual employers to decide whether they offer the vaccine. They may decide, for example, to limit the availability of the vaccine to exposed employees in ‘high risk’ groups such as smokers, older workers, or those in a clinical risk group. Of course, if fume exposure is not well controlled, employers cannot rely on the vaccine and must implement effective control measures. 

Most pulmonary function studies in welders show a significant decrease in FEV_1_ in smoking welders regardless of smoking status after 20/25 years of welding activity. In addition, a clear association between occupational exposure and COPD diagnosis has been reported. COPD, which represents a leading cause of premature death and disability in developed countries, is a relevantly susceptible condition for those workers who are occupationally exposed to WFs, making avoidance of direct and indirect exposure to tobacco smoke of primary importance. Therefore, it is crucial to stop smoking and to use collective and individual protection devices properly and, if necessary, to introduce control measures to reduce WFs exposure [95]. 

OA due to WFs is a key point since further exposure may affect OA prognosis for these workers; in the case of OA due to WFs, this type of occupation may have strict job limitations, putting their job at risk. For health surveillance purposes, specific questionnaires and lung function tests must be used to identify early symptoms and change in pulmonary function parameters. Furthermore, peak expiratory flow measurements must be carried out in suspected cases and, if necessary, to confirm diagnosis with a specific direct challenge test in such a way as to allow an immediate change of workplace tasks. 

This review has some limitations: (1) we limited our search to occupational disease and there may be a large proportion of welders who developed lung diseases after work life-long exposures to WFs where the link to the exposure is not clear on an individual level (i.e., sufficient to be recognized as an occupational disease), even when on a population level there would be a clear association (thus be labelled as work-associated). (2) We limited our review to lung diseases, despite the fact that metals from WFs are recognized to be one of the most critical components related to fine particulate on other health effects; in particular, fine PM is causing many more deaths due to cardiovascular diseases, such as heart infarction or stroke, than due to lung diseases.

Welders work with different materials under diverse conditions and are exposed to respiratory tract health hazards. The number of welders exposed to WFs is growing constantly despite process mechanization and automation. Acute respiratory injuries related to WFs are preventable with strict adherence to appropriate safety procedures. Reduction in welding exposure through engineering and correct use of personal protection will lead to a reduction in traditional welding-related respiratory diseases. However, with the development of new welding technologies, new hazards are likely to be introduced into the workplace. In particular, welding exposure is associated with small particles, e.g., NP production whose characterization and relationship with the development of human diseases is still unknown. NPs in WFs are considered a risk factor because of their specific particle characteristics, such as small size, large surface area, high particle concentrations, and complex metal composition. Studies on WFs in the area of particle research may aid the understanding of cellular and molecular mechanisms involved in welding-related lung carcinogenesis. 

Employers must implement primary prevention measures such as adequate ventilation systems, provide less hazardous welding materials, and use of welding processes to lower fume emissions. If possible, high-emission processes, such as MMAW and FCAW, should be replaced by low-emission processes, such as GMAW, GTAW, and SAW. Effective primary preventive measures, such as collective protective devices (CDPs) and personal protective equipments (PPEs), are also required. A well-designed air ventilation system coupled with good exhaust ventilation reduces exposure. Aspirator for fumes must be shaped and located in such a way as to be easily used. In confined spaces, where an adequate ventilation system is often not enough to ensure protection from WFs risks, personal protective respiratory devices must be utilized. Welders must be regularly informed and trained as to the importance of preventive measures, the abolition of cigarette smoke, and correct use of protective devices. Adherence for PPEs use depends on factors such as their ergonomic characteristics, the educational status of workers, and the organization of periodical information and training courses. WFs contain also NPs which may be even more dangerous than larger particles. What is more, inhaled particles from WFs create pulmonary inflammation and a subsequent systemic inflammatory response and this will increase the probability of atherosclerosis, followed by other cardiovascular events.

## 5. Conclusions

It has been demonstrated that pulmonary toxicity of complex metal-containing particulates can be associated with soluble forms of transition metals and dosage. Fumes generated during MMAW were found to be highly water soluble, whereas GMAW fumes were relatively insoluble. Greater emission fume processes, such as MMAW, cause an increased risk of pulmonary function abnormalities, COPD, lung cancer, etc., but even low-emission fume processes, such as GMAW and GTAW, produce high amounts of NPs, which are found to generate the highest ROS with longer lung retention compared to larger particles and are considered an additional risk factor for adverse health effects. Moreover, PM both in SS and MS WFs would seem to be a lung tumor promoter. Common chronic inflammatory respiratory diseases, like asthma, CB, and COPD are frequent in welders as a result of occupational exposure to WFs. Moreover, exposure to WFs increases the risk of infectious pneumonia and lung cancer. Ni, Zn, and Cr VI and toxic gases, such as O_3_ and NO_x_, may result in irritative and sensitizing respiratory tract effects. Zn, Cu, Mg, and Cd seem to be the principal causes of MFF. 

## Figures and Tables

**Table 1:** Type of electric arc welding process.

Electric arc Welding Process	Electrode	Shielding	Main Base Metal	Metal Oxides and Toxic Gases
Manual Metal Arc Welding (MMAW)	coated consumable electrode	decomposition of the electrode covering	all ferrous metals	Al, Cd, Co, Cr, Cu, Fe, fluorides, Mg, Mn, Mo, Ni, Pb, Si, Ti, Zn. Not high amounts of toxic gases: CO, CO_2,_ NO_x_, O_3_
Flux-cored Arc Welding (FCAW)	continuous filler metal consumable electrode	obtained from a flux contained within electrode	MS SS	Cr, Fe, fluorides, Mn, Ni, Si. Not high amounts of toxic gases: CO, CO_2_, NO_x_, O_3_,
Submerged Arc Welding (SAW)	uncoated consumable electrode	granulated flux (lime, Si, Mn oxide, calcium fluoride, and other compounds)	MS	Al, Fe, Fluorides, Mg, Mn, Si, Ti. Not high amounts of toxic gases: CO, CO_2_, NO_x_, O_3_,
Gas Metal Arc Welding (GMAW): Metal arc Inert Gas (MIG) Metal arc Active Gas (MAG)	continuous filler metal consumable electrode	MIG: inert gas (argon or helium) MAG: active gas (CO_2_)	Fe, MS, SS, Cu alloys, Ni alloys, Al, Mg	Al, Cr, Cu, Fe, Mn, Ni. High amounts of toxic gases: CO, CO_2_, NO_x_, O_3_
Gas Tungsten Arc Welding (GTAW)	tungsten non consumable electrode	inert gas (argon or helium)	Fe, SS, Al, Mg	Al, Cr, Fe, Mg, Mn, Ni. High amounts of toxic gases: CO, CO_2_, NO_x_, O_3_

Legend. MS: mild steel; SS: stainless steel; Al: aluminum; Cd: cadmium; Co: cobalt; Cr: chromium; Cu: copper; Fe: iron; Mn: manganese; Mg: magnesium; Mo: molybdenum; Ni: nickel; Si: silica; Pb: lead; Ti: titanium; Zn: zinc; CO: carbon monoxide; CO_2_: carbon dioxide; NO_x_: oxides of nitrogen; O_3_: ozone.

**Table 2:** Longitudinal study of lung function in welders.

Longitudinal Studies					
First Author, Publication Year Country	Years	Welders/Controls (*n*)	Welding Characteristics	Tests	Results
Mur, J.M., 1989, France [109]	1981/1986	138/106	Main base metals: MS, SS Welding processes: MIG	spirometry, CO transfer tests	No significant difference in spirometry and CO transfer test.
Chinn, D.J., 1990 UK [95]	1979/1986	286/64	Industry: shipyard Main base metals: MS Welding processes: MMA	Spirometry	Significant annual decline in FEV_1_ and FVC than controls
Beckett, W.S., 1996 USA [110]	3 year follow-up	24/35	Industry: shipyard Main base metals: SS Welding processes: TIG, MIG	Spirometry	No significant difference
Erkinjuntti-Pekkanen, R., 1999 New Zeeland [97]	1996/1998	43/35	Industry: engineering Main base metals: MS, SS Welding processes: MIG, TIG	Spirometry	No significant difference. In smoker group, welders had a significantly greater annual decline in FEV_1_ than non-welders
Christensen, S.W., 2008 Denmark [96]	1987/2004	68/32	Main base metals: SS, MS Welding processes: MMA, MAG, TIG	Spirometry	No significant differences. In smoker group, welders had a decline in FEV_1_ larger than non-welders while the difference was negligible in nonsmokers
Thaon, I., 2012 France [111]	1990/1995	543/709	Not specified	Spirometry	Significant decline in FEV_1_ in never smokers. Significant decline in FEV_1_ in nonsmokers welders exposed daily for ≥ 4 h/die but not in smokers
Haluza, D., 2014 Austria [98]	2002/2010	1326/NA	Not specified	Spirometry	Significant decline in FEV_1_ and FVC in heavily smoking welders (≥ 20 cigarettes/die) Significantly decrease of FEV_1_ and FVC associated with duration of exposure per year
Skoczyńska, A., 2016 Poland [101]	1980/2005	167/428	Not specified	Spirometry at the start of the employment and after 10, 20, and 25 years,	Significant difference In welders decrease in FEV_1_ and FVC after 20 years of exposure than controls. In welders decline of FEV_1_/year was greater compared to other workers.

Legend. FEV_1_: forced expiratory volume in 1 second; FVC: forced vital capacity; MS: mild steel; MMA: manual metal arc; SS: stainless steel; TIG: gas tungsten arc; MIG: metal arc inert; MAG: metal active gas; SS: stainless steel.

**Table 3:** Cross-sectional study of lung function in welders.

Longitudinal Studies					
First Author, Publication Year Country	Years	Welders/Controls (*n*)	Welding Characteristics	Tests	Results
Mur, J.M., 1985 France [112]	1985	346/214	Industry: factory producing industrial vehicles Main base metals: MS, SS Welding processes: Electric arc welding process	Spirometry, bronchial challenge test to acetylcholine, CO transfer tests	No significant difference in spirometry. Significant slightly higher bronchial hyper-reactivity to acetylcholine in welders than to controls Significant lower lung diffusing capacity for CO in welders than to controls
Ozdemir, O., 1995 Turkey [99]	Not specified	110/55	Working duration: 1-30y Main base metals: MS, Al Welding processes: MMA	Spirometry	Significant difference In smoker group FEV_1_, FVC and PEF were significantly lower in welders than controls.
Sobaszek, A., 1998 France [102]	Not specified	130/234	Industry: shipyards, tankbuilding Working duration ≥5y Welding metal: SS Welding processes: MMA, MIG, TIG	Spirometry before work shift	No significant difference The respiratory function of welders decreased after 25 years of SS welding activity and this decrease was not statistically linked to age but to time spent in welding.
Sobaszek, A., 2000 France [103]	2000	144 W (91SS, 43MS)/223 controls	Working duration ≥5y Main base metals: SS, MS Welding processes: MMA MIG TIG	Spirometry before and after work shift	Significant difference across-shift change in FVC and FEV_1_ between SS and MS and between SS and controls. SS welders with over 20 years of exposure had more significant across-shift decrease in FEV_1_, FVC, PEF than MS welders
Meo, S.A., 2003 Pakistan [100]	2003	50 W/50 C	Welding processes: MMA	Spirometry	Significant decrease in FEV_1_ and FEV_1_/FVC in welders with over 9 years of exposure compared to controls
Fidan, F., 2005 Turkey [108]	2004	34 W/20 C	Working duration ≥5year	Spirometry	FEV_1_/FVC across-shift significantly lower in welders than in controls

Legend. FEV_1_: forced expiratory volume in 1 second; FVC: forced vital capacity; PEF: peak expiratory flux; MS: mild steel; MMA: manual metal arc; SS: stainless steel; TIG: gas tungsten arc; MIG: metal arc inert; MAG: metal active gas; SS: stainless steel.

**Table 4:** Risk for lung cancer in welders. Standardized mortality ratio (SMR) and standardized incidence ratio (SIR): 95% confidence interval.

Longitudinal Studies						
First Author, Publication Year Country	Years	Welding Characteristics	Welders (*n*)	Observed	Expected	Standardized Mortality Ratio (SMR) Standardized Incidence Ratios (SIR)
Simonato, L., 1991 9 European countries [142]	1950–1980	Welding processes: MIG, MAG, TIG, MMA Main base metals: SS and MS	11.092	116	86.81	SMR = 1.34 (1.10 to 1.60)
Moulin, S.S., 1993 France [146]	1975–1988	Welding processes: MIG, MAG, TIG, MMA Main base metals: SS and MS	2.721	19	15.33	SMR = 1.24 (0.75 to 1.94)
Danielsen, T.E., 1993 Norway [154]	1940–1979	Welding processes: MIG, MAG, TIG, MMA Main base metals: SS and MS	623	9	3.6	SIR = 2.50 (1.14 to 4.75)
Hansen, K.S., 1996 Danish [145]	1964–1985	Welding processes: not specified Main base metals: SS and MS	6.180	51	36.84	SIR = 1.38 (1.03 to 1.81)
Steenland, K., 2002 United States [149]	1988–1998	Welding processes: Not specified Main base metals: SS and MS	4.459	108	73.97	SMR = 1.46 (1.20 to 1.76)
Sørensen, A.R., 2007 Danish [151]	1968–2003	Welding processes: MAG, TIG, MMA Main base metals: SS and MS	4.539	75	55.4	SIR = 1.35 (1.06 to 1.70)

Legend: MMA: manual metal arc; SS: stainless steel; TIG: gas tungsten arc; MIG: metal arc inert; MAG: metal active gas; SS: stainless steel.

**Table 5:** Risk for lung cancer in welders: Largest recent case-control studies.

Largest Recent Case-Control Studies				
First author, Publication Year Country	Years	Welders/Controls (*n*)	Welding Characteristics	Odds Ratio (95% Confidence Interval)
t Mannetje, A., 2012 15 centers in Central and Eastern Europe and the UK [144]	1998–2001	296/247	Welding processes: Arc and gas welding Main base metals: SS and MS	1.22 (0,99 to 1,50) ° 1.13 (0,90 to 1.43) * 1.38 (1.00 to 1.90) **
Kendzia, B., 2013 SYNERGY project: 16 studies conducted in Europa, Canada, China and New Zeland [160]	1985–2010	568/427	Industry: Shipbuilding and repair Construction Manufacture of machine Manufacture of motor vehicles and motor bike	1.44 (1.25 to 1.67) ^a^ 1.77 (1.31 to 2.39) ** 1.58 (1.32 to 1.89) ^b^ 1.41 (1.09 to 1.82) ^c^
Matrat, M., 2016 France [163]	2001–2007	92/64	Welding process: Soldering, Brazing, Gas welding, Arc welding, Spot welding, Other welding	1.7 (1.1 to 2.5) ° 2.0 (1.0 to 3.9) ^d^

Legend: MS: mild steel; SS: stainless steel; ° adjusted for age, center, education, smoking, and asbestos; * after adjustment for welding-related chromium exposure; ** after 25 years of exposure; ^a^ adjusted for age, center, education, smoking; ^b^ for squamous cell carcinoma (SqCC); ^c^ for small cell lung cancer (SCLC); ^d^ 10–20 years since last welding.
